# Effects of telitacicept and belimumab on systemic lupus erythematosus: a systematic review and meta-analysis

**DOI:** 10.1038/s41598-025-29929-9

**Published:** 2025-11-27

**Authors:** Jiawen Qian, Xiujie Lv, Yuxin Hu, Yuxin Zhang, Rong Zhou, Chenyi Yuan, Zhehao Huang, Xiangfu Gao, Yuan Gao, Yuancheng Gao

**Affiliations:** 1 The First Affiliated Hospital of Zhejiang Chinese Medical University, Hangzhou, 310000 China; 2 Dongzhimen Hospital, Beijing University of Chinese Medicine, Beijing, 101121 China; 3 The Second Affiliated Hospital of Zhejiang Chinese Medical University, Hangzhou, 310021 China; 4 Changxing County Hospital of Traditional Chinese Medicine, Huzhou, 313100 China; 5 The First Affiliated Hospital of Ningbo University, Ningbo, 315000 China; 6 Capital Medical University, Beijing, 100069 China

**Keywords:** Diseases, Immunology, Medical research, Rheumatology

## Abstract

B cell-targeted therapies play an important role in systemic lupus erythematosus (SLE). Belimumab targets B lymphocyte stimulator (BLyS), whereas telitacicept inhibits both BLyS and APRIL. Although both drugs have demonstrated clinical efficacy in SLE, comparative benefits and risks of telitacicept versus belimumab remain unclear. We performed a systematic review and meta-analysis using indirect comparisons to compare their efficacy and safety. We searched 6 database for randomized controlled trials (RCTs) published up to November 1, 2025, without language restriction. Trials evaluating belimumab or telitacicept in adult SLE patients were included. Primary outcomes included SLE Responder Index 4 (SRI4), SRI7 response rates and decreasing SLEDAI score. Secondary outcomes included prednisone dose reduction, anti-dsDNA change, adverse events (AEs) and serious AEs. Data extraction and risk-of-bias assessment were performed independently by two reviewers. Analysis used RR with 95% CI, and heterogeneity was assessed by I^2^. Sensitivity, subgroup and publication-bias analyses were performed. 11 trials with telitacicept and belimumab involving 4303 participants were included. Compared with the belimumab group, telitacicept significantly increased the SRI4 response rate (relative risk [RR], 2.03, 95%CI, 1.65–2.49, *p* < 0.0001), SRI7 response rate (RR, 3.61, 95%CI, 1.57–8.29, *p* = 0.002) and decreased SLEDAI score (RR, 1.67, 95%CI, 1.41–1.97, *p* < 0.0001). Compared with belimumab, telitacicept exhibited a significant advantage in SRI4 response rate (p for interaction = 0.0002), without a significant difference in adverse events. Certainty of evidence ranged from moderate to high, but heterogeneity was present for some outcomes. Telitacicept improved SRI4 and SRI7 response rates, reducing disease activity and prednisone dosage, without a clear increase in infection risk compared with belimumab. Dual inhibition of BLyS and APRIL by telitacicept may offer an effective option for reducing SLE activity. Further large-scale, long-term head-to-head trials are needed to confirm these findings.

**Supplementary Information:**

The online version contains supplementary material available at 10.1038/s41598-025-29929-9.

## Introduction

Systemic lupus erythematosus (SLE) is a chronic systemic autoimmune disease. The global SLE incidence and newly diagnosed population were estimated to be 5.14 (1.4 to 15.13) per 100,000 person-years and 0.40 million people annually^1^. The pathogenesis of SLE is that the immune system attacks its tissues, causing widespread inflammation and tissue damage in various affected organs. Treating and identifying novel therapies for lupus is challenging because of its genetic and phenotypic heterogeneity^2^. Also, there is pathophysiological heterogeneity in SLE patients, including interferon signature, autoantibody profiles, neutrophil extracellular traps, and T-cell abnormalities^3,4^. These heterogeneities not only make it difficult for treatment decisions and prognostication, and have made drug development quite challenging. Established therapies are broad-spectrum, including hydroxychloroquine, corticosteroids, and immunosuppressive agents^2,5,6^. However, long-term use of the drugs can lead to some side effects of retinopathy, documented skin hyperpigmentation, and even infections, hypertension, and osteoporosis^7–9^. To precisely improve clinical efficacy while reducing adverse events, trials on B-cell targeted drugs have been established.

B cell-targeted therapies have been established and approved, enhancing our armamentarium of traditional therapies^2^. Over-activation of B cells leads to immune imbalance, which is the main pathogenesis of SLE. Therefore, B cells have been major targets for developing new therapies for SLE.

Belimumab is a classic B lymphocyte stimulator (BLyS)-targeting monoclonal antibody. It effectively decreased BLyS levels in patients with SLE correlated with disease activity, thus has a wide clinical usage^10,11^. Telitacicept provides a novel option for patients with active SLE. As a dual-targeting drug, telitacicept decreases A proliferation-inducing ligand (APRIL) and BLyS serum levels. APRIL is a molecule involved in activating B cells, it was active in the later stage of B-cell activation, which regulates the function and survival of effector B cells. Elevated levels of BLyS and APRIL are frequently observed in SLE. Telitacicept exhibits a unique structure, inhibiting the function of BLyS and APRIL, decreasing antibody formation and disease activity^12^.

Compared with belimumab, telitacicept has a more effective immunologic suppression mechanism, whereas the benefits and limitations between the two drugs remain unclear. As multiple clinical trials have been conducted to evaluate several outcomes of telitacicept and belimumab, we performed the first systematic review and meta-analysis of randomized controlled trials (RCTs) for a quantitative comparison between the two drugs. Because of the lack of direct head-to-head RCTs, we utilized indirect comparisons to provide preliminary evidence of comparability. It provides a new perspective on the management of SLE.

## Methods

### Data sources, search strategy

We performed this analysis following the Preferred Reporting Items for Systematic Reviews and Meta-analysis (PRISMA) guidelines and Cochrane Handbook, with the method of indirect comparison recommended in Chap. 11.2. Indirect comparison means that the difference between the summary statistics of the intervention effect in the direct A versus C and A versus B meta-analysis provides an indirect estimate of the B versus C intervention effect^13^.

We registered in PROSPERO (registration CRD42023392842). We systematically searched PubMed, Web of Science, MedLine, Embase, Cochrane Library, and Clinicaltrials.gov for articles published before November 1, 2025, with no language restriction. Searching terms including “Systemic Lupus Erythematosus”, “telitacicept” and “belimumab”. The full search strategy is provided in the Supplementary File.

### Study selection

#### Inclusion criteria

All included studies must meet the following eligibility criteria: (1) RCTs; (2) investigation of the effect of belimumab or telitacicept; (3) investigations on adult SLE patients (age ≥ 18); (4) estimation of the relative risk using standardized incidence ratio, relative risk (RR), hazard ratio (HR), odds ratio (OR) or incidence rate ratios (IRR) with corresponding 95% confidence interval (CI).

#### Exclusion criteria

The criteria for excluding selected studies from the meta-analysis are as follows: (1) non-RCTs; (2) studies were excluded if the interventions were other than telitacicept or belimumab; (3) not conducted in SLE patients; (4) studies without a description of including SLE disease activity index (SLEDAI) criteria; (5) duplicated or unfinished studies; (6) studies conducted in patients age < 18.

### Data extraction and quality assessment

Study searching, data exclusion, and quality assessment were performed by two researchers (QJW and HYX) independently. The third researcher was consulted if a disagreement was met (LXJ). Full articles and published reports were obtained, and relevant data was extracted. Data extraction included the following information: title, author, disease, Inclusion criteria, number of patients, average age, gender, study type, intervention measures, course of treatment, outcome indicators, follow-up results, and adverse events (AEs). The first outcome include SRI4 response rate, SRI7 response rate, and rate of SLEDAI score decreasing. The second outcome include prednisone dose reduction, anti-dsDNA decreasing, AEs, and serious adverse events (SAEs). SRI4 stands for the proportion of patients who meet the SLE Responder Index (SRI) criteria with at least a 4-point reduction in SLEDAI score, no new British Isles Lupus Assessment Group (BILAG) A, ≤ 1 new BILAG B, and no meaningful worsening on the Physician Global Assessment (PGA). SRI7 stands for at least a 7-point reduction in SLEDAI instead of 4, with a same requirement for BILAG and PGA. We assessed the study quality with Review Manager 5.4 (Cochrane Collaboration, Oxford, UK).

### Risk of bias assessment

The risk of bias was assessed following the Cochrane quality criteria. Two evaluators reached a consensus on aspects: random sequence generation, allocation concealment, blinding, reporting bias, attrition bias, and any other potential sources of bias. Each trial was rated as low risk, unclear risk, and high risk of bias. A third evaluator adjudicated any disagreements. The results of the assessments were summarized and visualized with Review Manager 5.4.

### Data synthesis and analysis

Meta-analysis was performed. When values of the outcomes were reported as median and interquartile range (IQR), the conversion of mean and standard deviation (SD) was adopted based on the Cochrane Handbook for Systematic Reviews of Interventions^13^. Weighted mean difference (WMD), relative risk (RR), and 95% confidence interval (CI) were utilized as effect indicators. A random-effects model was established if I^2^ > 50% or *p* < 0.05, and a fixed-effects model was established if I^2^ ≤ 50% or *p* ≥ 0.05. Heterogeneity was analyzed with chi^2^, I^2^, and p was calculated as effect indicators. I^2^ values of 25%, 50%, and 75% corresponded to cutoff points for low, moderate, and high degrees of heterogeneity^14^.

Sensitivity analysis was conducted to assess the robustness of the results. Subgroup analysis was performed to detect possible sources of heterogeneity. Publication bias was assessed with a funnel plot and Egger’s test. Trim-and-fill was applied when appropriate. Statistical significance was defined as *p* < 0.05.

## Results

**Fig. 1:**
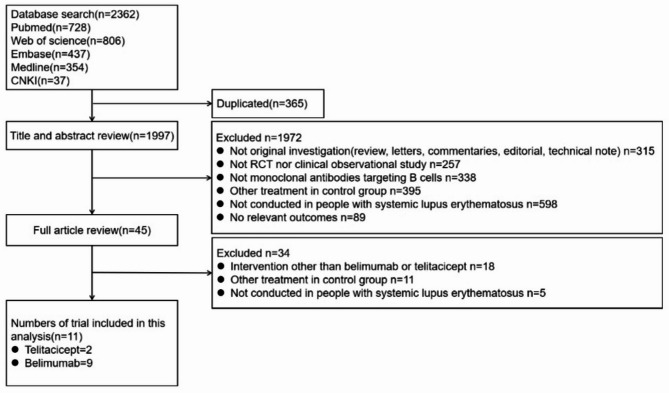
Diagram of the identification process for eligible studies. RCT, randomized controlled trial.

This research yielded 2362 articles, and 11 trials with 4303 patients were finally included (Fig. 1).

### Study characteristics

Of all the RCT trials included, 9 trials involved belimumab as an invention, and 2 trials involved telitacicept. Eleven trials enrolled 2486 patients in the experimental group and 1817 in the control group, most of which were females. Patients of all the trials are adults, with a mean of age from 32 to 42. SLE disease duration of patients varied from 5 to 14 years in adults. Subcutaneous injection was utilized in 3 trials, and 8 trials utilized intravenous administration. Basic SELENA-SLEDAI score varied from 3 to 12, 6 with an inclusion criterion of SLEDAI ≥ 8, 2 trials ≥ 6, and 1 trial ≥ 4. In the trial by Furie et al. (2008), the baseline SLEDAI score ranged from 2 to 4, although no specific inclusion criterion for SLEDAI was reported. (Table 1).

**Table 1:** Characteristics of included trials.

Study	Type of study	Follow-up duration, weeks	Intervention		Patients, *n*		Gender, female		Age, years		Disease duration, years		Inclusion SLEDAI criteria	SELENA-SLEDAI	
			T	C	T	C	T	C	T	C	T	C		T	C
Furie 2008	RCT	15 weeks	Belimumab	Placebo	14	13	12	11	40.28 ± 32.13	42.38 ± 23.26	9.94 ± 15.65	7.16 ± 12.38	NA	3.46 ± 6.59	2.54 ± 3.32
Furie 2011	RCT	76 weeks	Belimumab	Placebo	273	275	259	252	40.5 ± 11.1	40 ± 11.9	7.9 ± 7.1	7.4 ± 6.7	>6	9.5 ± 3.6	9.8 ± 4.0
Navarra 2011	RCT	52 weeks	Belimumab	Placebo	290	287	280	270	35.4 ± 10.8	36.2 ± 11.8	5.0 ± 5.1	5.9 ± 6.2	≥ 6	10.0 ± 3.9	9.7 ± 3.6
Wallace 2009	RCT	52 weeks	Belimumab	Placebo	111	113	105	102	41.8 ± 11.7	42.2 ± 10.9	8.5 ± 8.0	8.1 ± 7.4	>4	9.5 ± 0.4	9.5 ± 0.5
Stohl 2017	RCT	52 weeks	Belimumab	Placebo	556	280	521	268	38.1 ± 12.1	39.6 ± 12.61	13.55 ± 26.01	14.70 ± 28.32	≥ 8	10.5 ± 3.19	10.3 ± 3.04
Zhang 2018	RCT	52 weeks	Belimumab	Placebo	451	226	419	210	32.3 ± 9.65	31.7 ± 9.18	6.07 ± 5.04	5.97 ± 5.19	≥ 8	9.8 ± 3.83	10.2 ± 4.11
Tanaka 2018	RCT	52 weeks	Belimumab	Placebo	39	21	35	20	38.1 ± 10.23	33.7 ± 10.61	8.4 ± 6.34	10.4 ± 6.37	≥ 8	10.1 ± 2.82	10.3 ± 3.16
Furie 2020	RCT	104 weeks	Belimumab	Placebo	223	223	197	196	33.7 ± 10.7	33.1 ± 10.6	12.5 ± 5.3	12.2 ± 4.8	NA	12.5 ± 5.3	12.2 ± 4.8
Ginzler 2022	RCT	52 weeks	Belimumab	Placebo	299	149	290	144	38.6 ± 11.1	39.3 ± 12.2	7.3 ± 7.08	6.9 ± 7.38	≥ 8	9.9 ± 3.52	10.2 ± 2.90
Di 2023	RCT	48 weeks	Telitacicept	Placebo	63	62	61	58	33.5 ± 10.3	34.9 ± 9.6	6.67 ± 5.21	8.79 ± 5.87	≥ 8	NA	NA
Vollenhoven 2025	RCT	52 weeks	Telitacicept	Placebo	167	168	152	157	34.8 ± 9.8	35.1 ± 10.4	7.5 ± 5.5	7.2 ± 5.3	≥ 8	11.5 ± 3.1	11.5 ± 3.6

### Risk of bias

Of the included trials, 7 trials were evaluated as low risk of bias, 3 trials were evaluated as unknown risk of bias, and 1 trials were evaluated as high risk of bias. Most of the unknown risks of selection bias result in an absence of full description, blinding of participants and personnel was unclear in 3 trials, and blind of outcome assessment was unclear in 2 trials. 10 trials (90.9%) were reported to be randomized, 4 trials (36.36%) had clear allocation concealment, and 8 trials 72.73%) reported complete blinding of participants and personnel. All the trials described clearly the outcomes. No trial had a risk of selective reporting or a risk of incomplete outcome data. Other bias was low in all the 11 trials. (Supplementary Fig. 1–2)

### Primary outcomes

#### Effects on SRI4 response rate

**Fig. 2:**
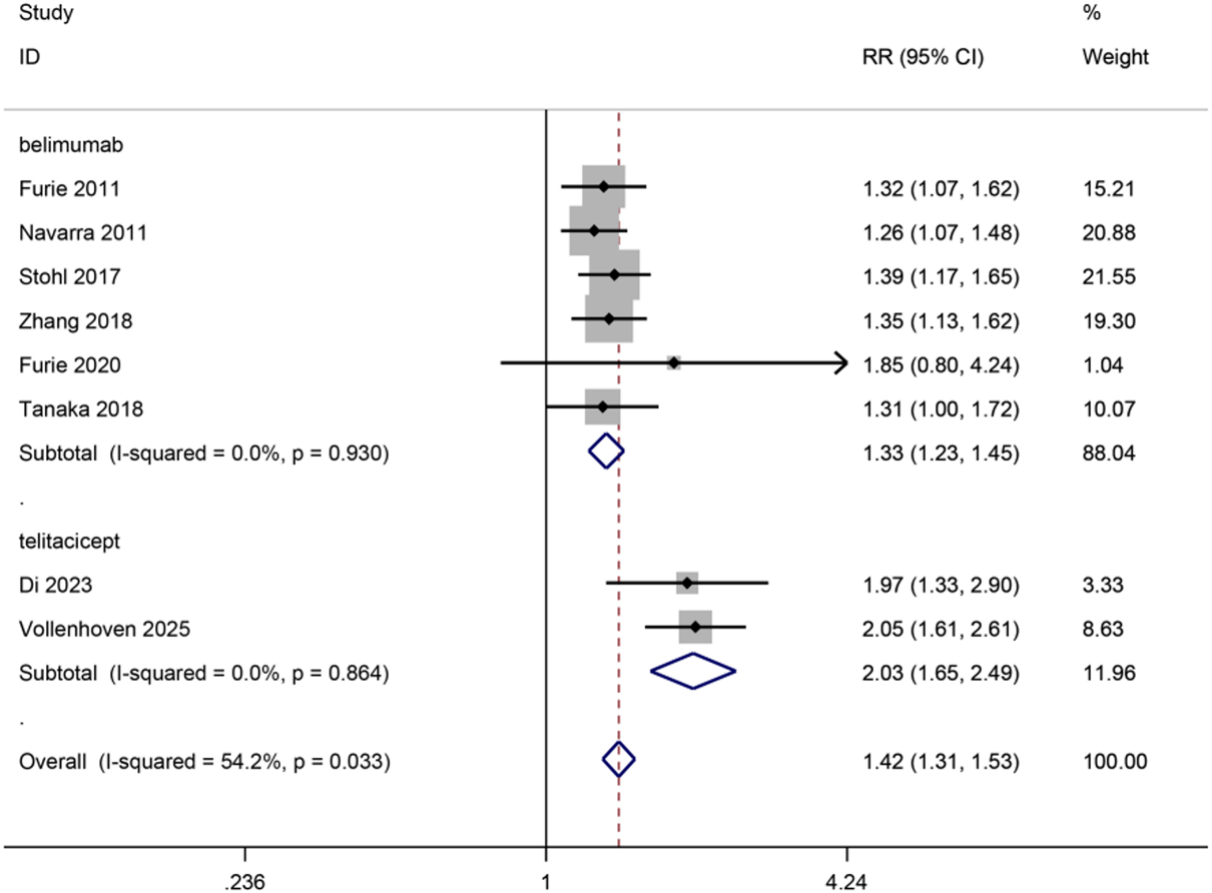
Diagram of the SRI4 response rate for eligible studies. SRI, the Systemic Lupus Erythematosus Responder Index.

Data related to the effects of drugs on SRI4 was found in 8 trials, 6 belimumab trials, and 2 telitacicept trials, with 3632 patients included. Both belimumab and telitacicept increased the SRI4 response rate. Telitacicept significantly increased the SRI4 response rate (2 trials, RR, 2.03, 95%CI, 1.65–2.49, *p* < 0.0001, moderate certainty) while belimumab exhibited a weaker effect (6 trials, RR, 1.33, 95%CI, 1.23–1.45, *p* < 0.0001, high certainty), with a significant difference between drugs (p for interaction = 0.0002 < 0.05, I^2^ = 92.8%). (Fig. 2)

#### Effects on SRI7 response rate

Data related to the effects of drugs on SRI7 was found in 2 trials, 1 belimumab trial and 1 telitacicept trial, with 185 patients included.Telitacicept increased the SRI7 response rate (1 trial, RR, 3.61, 95%CI, 1.57–8.29, *p* = 0.002, high certainty), while belimumab was not significant (1 trial, RR, 1.39, 95%CI, 0.30–6.52, *p* = 0.68, high certainty). Significant difference was not observed between drugs (p for interaction = 0.29, I^2^ = 12.1%). (Supplementary Fig. 3)

### Effects on SLEDAI score

**Fig. 3:**
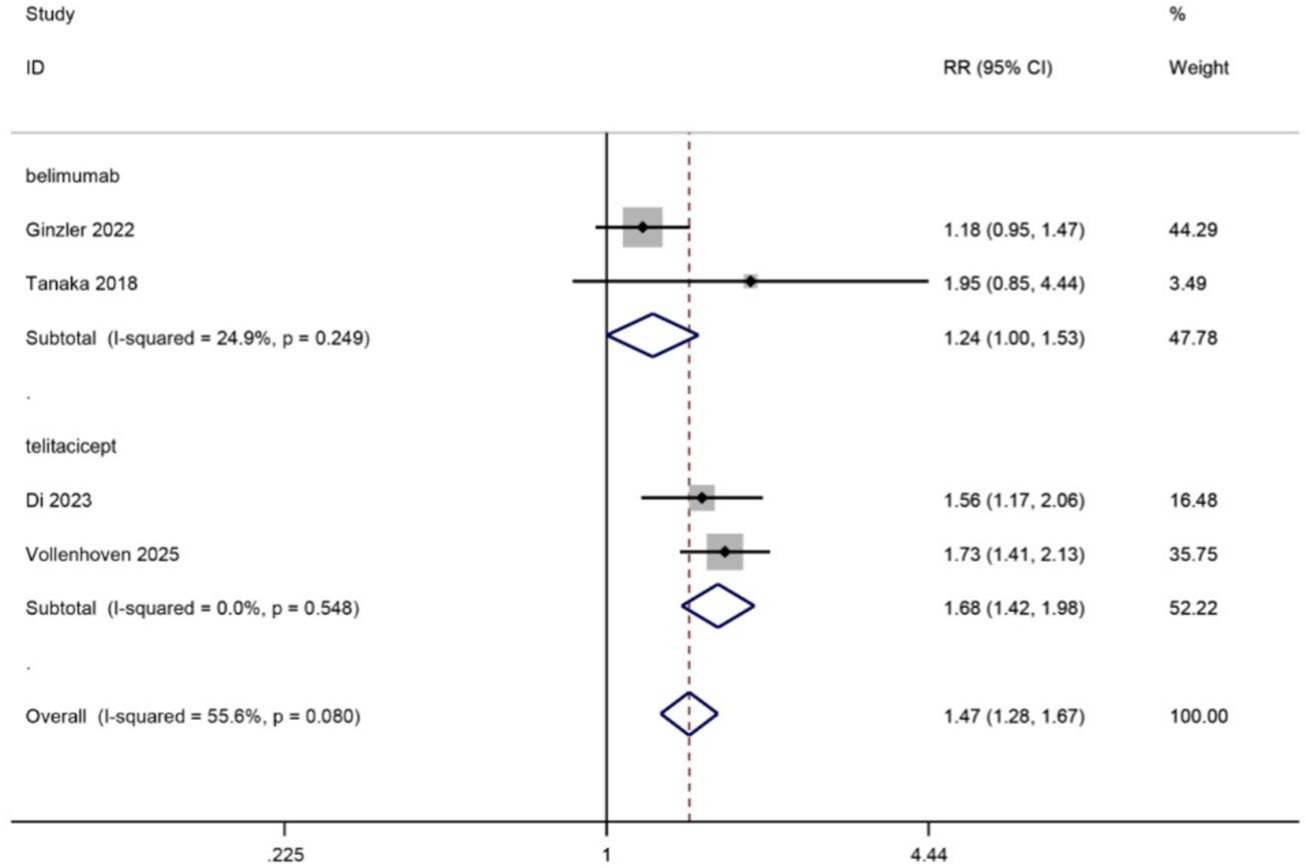
Diagram of the SLEDAI score for eligible studies.

Data related to the effects of SLEDAI score decrease was found in 4 trials, 2 belimumab trial and 2 telitacicept trial, with 966 patients included. Both belimumab and telitacicept decreased SLEDAI score, and telitacicept exhibits a significant effect (belimumab: 2 trials, RR, 1.29, 95%CI, 0.89–1.87, *p* = 0.18, high certainty; telitacicept: 1 trial, RR, 1.67, 95%CI, 1.41–1.97, *p* < 0.0001, high certainty). Significant difference was not observed between drugs (p for interaction = 0.22, I^2^ = 33.6%). (Fig. 3)

### Secondary outcomes

#### Effects on prednisone dose reduction

Data related to the effects of drugs on prednisone dose reduction was found in 8 trials, 6 belimumab trials, and 2 telitacicept trials, with 2427 patients included. Belimumab decreased the prednisone dose reduction with a significant effect (belimumab: 6 trials, RR, 1.49, 95%CI, 1.21–1.84, *p* < 0.0001, high certainty), while telitacicept exhibited a weaker effect (telitacicept: 2 trials, RR, 1.26, 95%CI, 0.98–1.63, *p* = 0.07, moderate certainty). No significant difference was observed between drugs (p for interaction = 0.33, I^2^ = 0%). (Supplementary Fig. 4)

#### Effects on anti-dsDNA

Data related to the effects of drugs on anti-dsDNA was found in 4 trials, 3 belimumab trials, and 1 telitacicept trial, with 1038 patients included. Both belimumab and telitacicept decreased anti-dsDNA, belimumab exhibited a significant effect (3 trials, RR, 2.52, 95%CI, 1.57–4.07, *p* < 0.0001, high certainty), while a significant effect was not observed in telitacicept (1 trial, RR, 6.04, 95%CI, 0.73–49.59, *p* = 0.09, high certainty). However, there was no significant difference between drugs (p for interaction = 0.43, I^2^ = 0%). (Supplementary Fig. 5)

### Safety outcomes

#### AEs

**Fig. 4:**
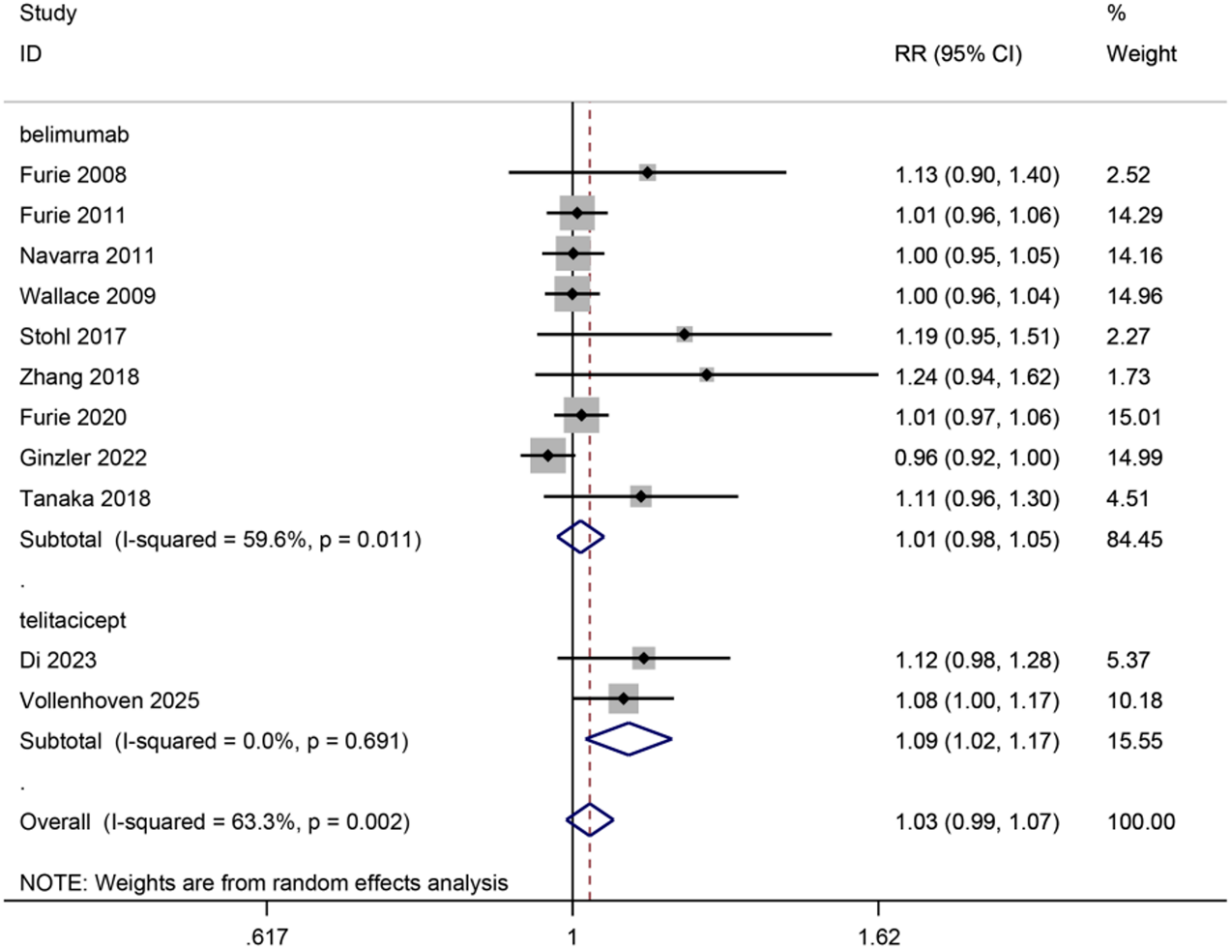
Diagram of the adverse events for eligible studies.

Overall, 11 trials reported data about AEs, 9 belimumab trials, and 2 telitacicept trials, with 4376 patients included. Telitacicept exhibited a significant effect on the occurrence of AEs (2 trials, RR, 1.09, 95%CI, 1.02–1.17, *p* = 0.01, moderate certainty), while belimumab was not significant (9 trials, RR, 1.03, 95%CI, 1.00-1.06, *p* = 0.07, moderate certainty). There was no significant difference between drugs (p for interaction = 0.12, I^2^ = 58.0%). (Fig. 4)

#### Serious AEs

**Fig. 5:**
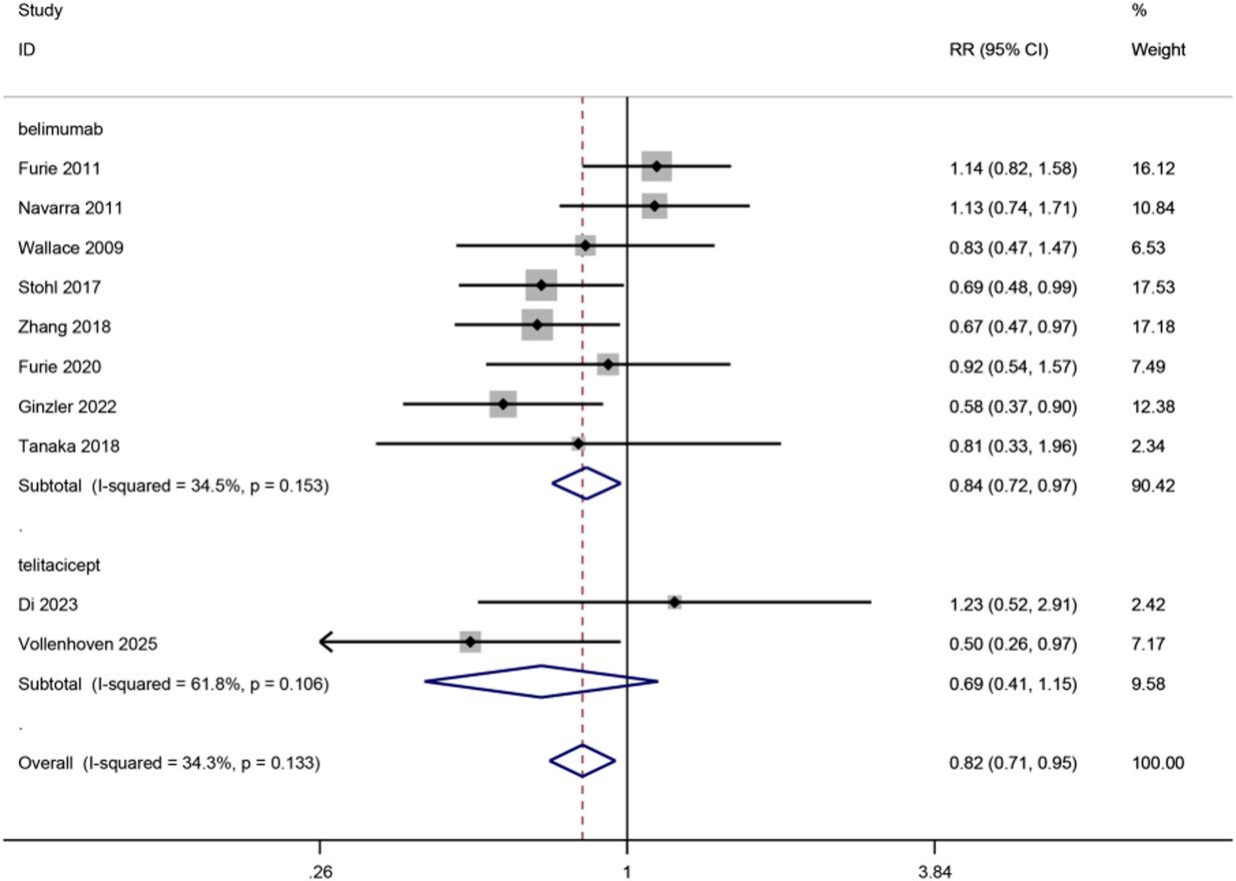
Diagram of serious adverse events for eligible studies.

10 trials reported data on drugs’ effects on serious AEs, 8 belimumab trials, and 2 telitacicept trials, with 4305 patients included. Belimumab exhibited a significant effect on the occurrence of serious AEs(8 trials, RR, 0.84, 95%CI, 0.72–0.97, *p* = 0.02, moderate certainty), while telitacicept was not significant (2 trials, RR, 0.69, 95%CI, 0.41–1.15, *p* = 0.09, high certainty). Significant difference exhibits between drugs (p for interaction = 0.34, I^2^ = 0%). (Fig. 5)

### Specific AEs and deaths

#### Risk of infections and infectious diseases

We launched analysis on different specific AEs. 10 trials reported data related to specific AEs of infections and contagious diseases, 8 belimumab trials, and 2 telitacicept trials, with 3138 patients included. Neither the drugs exhibited significant effect on the incidence rate of infections and infectious diseases (telitacicept: 2 trials, RR, 1.25, 95%CI, 0.99–1.57, *p* = 0.06, moderate certainty; belimumab: 8 trials, RR, 1.03, 95%CI, 0.96–1.09, *p* = 0.42, moderate certainty). There was no significant difference between two drugs (p for interaction = 0.11, I^2^ = 60.0%).(Supplementary Fig. 6).

#### Risk of general disorders and administration site conditions

5 trials reported data related to specific AEs of general disorders and administration site conditions, 3 belimumab trials, and 2 telitacicept trial, with 1809 patients included. Neither the drugs exhibited a significant effect on the incidence rate of infections and infectious diseases (belimumab: 3 trials, RR, 1.00, 95%CI, 0.79–1.26, *p* = 0.99, moderate certainty; telitacicept: 2 trials, RR, 6.75, 95%CI, 0.85–53.62, *p* = 0.07, high certainty). There was no significant difference between two drugs (p for interaction = 0.07, I^2^ = 69.0%). (Supplementary Fig. 7)

#### Risk of other AEs and deaths

Despite infections and AEs caused by administration, one case of hypersensitivity reaction and one case of drug hypersensitivity are reported in telitacicept studies. Musculoskeletal and connective tissue disorders (11.68%), gastrointestinal disorders (22.05%), nervous system disorders (19.35%), and hematological system disorders (16.11%) are reported in belimumab studies. 9 belimumab trials reported deaths, including 30 cases, 17 cases in the experimental group, and 13 cases in the control group. There is no significant heterogeneity among trials (*p* = 0.78, I^2^ = 0%). (Table 2).

**Table 2:** Results of safety outcomes.

Outcome	k	*n*	Telitacicept	Belimumab	*p* for interaction	I^2^
AEs	11	4376	1.09 (1.02, 1.17)	1.03 (1.00, 1.06)	0.12	58.00%
Serious AEs	10	4305	0.69 (0.41, 1.15)	0.84 (0.72, 0.97)	0.34	0%
Risk of infections and infectious diseases	10	3138	1.25 (0.99, 1.57)	1.03 (0.96, 1.09)	0.11	60.00%
Risk of general disorders and administration site conditions	5	1809	1.00 (0.79, 1.26)	6.75 (0.85, 53.62)	0.07	69.00%

### Subgroup analysis

#### Subgroup analysis of SRI4 response rate

Although the SRI4 response rate was significantly different between the two drugs, a high heterogeneity of 92.8% was observed in the study. As a result, we launched subgroup analysis on disease duration, sample size of trials, and injection methods of drugs. (Supplementary Fig. 8)

In large sample size trials (sample size ≥ 30), both telitacicept showed a significant increase in SRI4 response rate, while small size trials exhibited no significant effect, indicating that sample size might be a source of heterogeneity. Both belimumab and telitacicept increased the SRI4 response rate with subcutaneous injection. Subcutaneous injection trials were more effective than intravenous injection in belimumab, but the difference was not significant. Different disease duration did not lead to differences in SRI4 response rates between belimumab and telitacicept.

### Publication bias

Funnel plots and Egger’s test were performed to assess the possibility of publication bias. Funnel plots were symmetric, indicating no evidence of a significant publication bias. SRI4 response rate, prednisone dose reduction, and serious AEs have significant evidence of publication bias and small-study effects (Egger’s test, *p* < 0.05). After trimming and filling, publication bias had no significant effect on these outcomes, thus the output of our analysis may be considered stable. (Supplementary Fig. 9)

### Sensitivity analysis

To demonstrate the robustness of the results, the gradual elimination method was adopted for sensitivity analysis. After leave-one-out test, no obvious directional change occurred in the other results, indicating the robustness of the synthesized results. (Supplementary Fig. 10)

## Discussion

The discussion must not contain subheadings. Present study consolidated data from extensive trials of telitacicept and belimumab for SLE patients, indicating that in the SLE patients, telitacicept and belimumab, increased SRI4 response rate, SRI7 response rate, whereas decreasing SLEDAI score and prednisone dose, decreased disease activity of SLE. According to current data, there is no significant difference in AEs between telitacicept and belimumab.

### Effects and safety of telitacicept and Belimumab in SLE

The current data indicated that telitacicept increased SRI4 and SRI7 response rates in SLE patients, reduced disease activity, and alleviated symptoms such as rash and joint pain, thus enhancing the life quality of SLE patients.

Moderate certainty evidence suggested that the SRI4 response rate was significantly higher in the telitacicept group. The result is consistent with the previous study. Despite telitacicept, atacicept, another BLyS / APRIL inhibitor launched by Merck, also showed a high SRI4 response rate of 53.8% to 57.8% compared to 44.0% in the placebo group^15,16^. The SRI7 response rate also increased significantly within telitacicept trials, while a significant increase was not observed in belimumab trials.

Anti-dsDNA is one of the anti-DNA antibodies. High certainty evidence suggested that belimumab significantly decreased anti-dsDNA, while telitacicept had no significant effect. SRI response rates and anti-dsDNA are important parameters for SLE disease activity, whereas anti-dsDNA results are not consistent with SRI response rates. Anti-dsDNA is an important indicator of SLE disease activity and is widely used in clinical practice. Still, as a single laboratory indicator, it is affected by various factors such as test methods, sample collection, etc. The SRI response rate includes three important clinical scores, namely the PGA score, the BILAG score, and the SLEDAI score, which are more cumbersome to evaluate and more stringent in terms of requirements. Compared with anti-dsDNA, the SRI response rate can reflect patients’ overall status in a more comprehensive and detailed way, so we believe that the results of the SRI response rate are more reliable. The SRI4 response rate requires a decrease of 4 or more SLEDAI scores from baseline, while the SRI7 response rate requires a decrease of 7 or more SLEDAI scores from baseline, which is more stringent than SRI4. In our study, telitacicept had a significant advantage over belimumab in the SRI4 response rate. In contrast, in the SRI7 response rate, telitacicept had a significant effect compared to placebo, but not a significant effect compared to belimumab. This may be because telitacicept was less effective in patients with relatively high SLEDAI scores and severe clinical symptoms, such as lupus encephalopathy. Further clinical trials are needed to demonstrate the efficacy of telitacicept in severe lupus.

Prednisone is widely utilized in the clinical treatment of SLE, and reducing prednisone dose is a key treatment goal in SLE^17^. Previous studies suggested that both telitacicept and belimumab may have the potential to reduce prednisone use^12,18^. Our analysis demonstrated that telitacicept was not inferior to belimumab. Reducing the prednisone dose can help doctors better balance treatment and side effects, reducing the probability of side effects such as mood swings, weight gain, and blood sugar elevation, and enhancing patients’ life quality. Telitacicept may reduce the psychological burden caused by long-term use of prednisone, improving compliance of patients, and thereby achieving stable treatment effects. Long-term prednisone usage in SLE patients may also cause a high risk of severe complications, including diabetes and cardiovascular diseases^19^. Decreasing prednisone usage can prevent complications from occurring.

Cases of telitacicept in the treatment of refractory membranous nephropathy (MN) have been reported^20^. A strong association between high levels of APRIL and renal activity was also found in a study of patients with SLE^21^. A multicentre real-world study conducted by Jin also reported improvement in renal function after treatment with telitacicept^22^. Currently, there is no trial specifically evaluating telitacicept in lupus nephritis patients, thus its effects on renal function require further investigation.

A previous clinical study of atacicept, another APRIL inhibitor, showed two deaths due to infection in the 160 mg dosing group, suggesting that APRIL inhibition on top of BLyS may lead to hyperimmunosuppression, which increases the risk of infection^23^. Belimumab also carries the risk of causing infections, with the most commonly reported infections in belimumab studies including upper respiratory tract infections, gastrointestinal disorders, and other infectious diseases. Gastrointestinal disorders, and musculoskeletal and connective tissue disorders, whereas the most commonly reported infections in telitacicept studies included upper respiratory tract infections, urinary tract infections, and shingles. We performed a meta-analysis of both AEs and infection-related AEs in the belimumab and telitacicept 160 mg dosing groups, and the results suggested that there were no significant differences between the two drugs. However, the safety data of telitacicept is based on a small number of trials, with preliminary evidence showing no differences, more data is needed. More high-quality long-term follow-up studies are needed to demonstrate the long-term safety of telitacicept.

High heterogeneity was observed in the analysis of the SRI4 response rate. We performed subgroup analysis to identify heterogeneity sources. The results indicated that the SRI4 response rate might be related to sample size.

We assessed the bias with the GRADE score. Several studies we included had small sample sizes and may have publication bias. We performed the sensitivity analysis to minimize the impact on the results. However, there are still risks in corrected results. Further large-sample, multi-center studies with high-quality evidence are needed to verify the accuracy of the results.

Unlike the traditional administration of belimumab, telitacicept exhibits a more convenient way of subcutaneous injection, while it may lead to a potentially high AE rate. Results showed no significant heterogeneity among subgroups of different injection methods in AEs, while the report of AEs may be limited by the number of included patients.

### Potential mechanisms of telitacicept and Belimumab

**Fig. 6:**
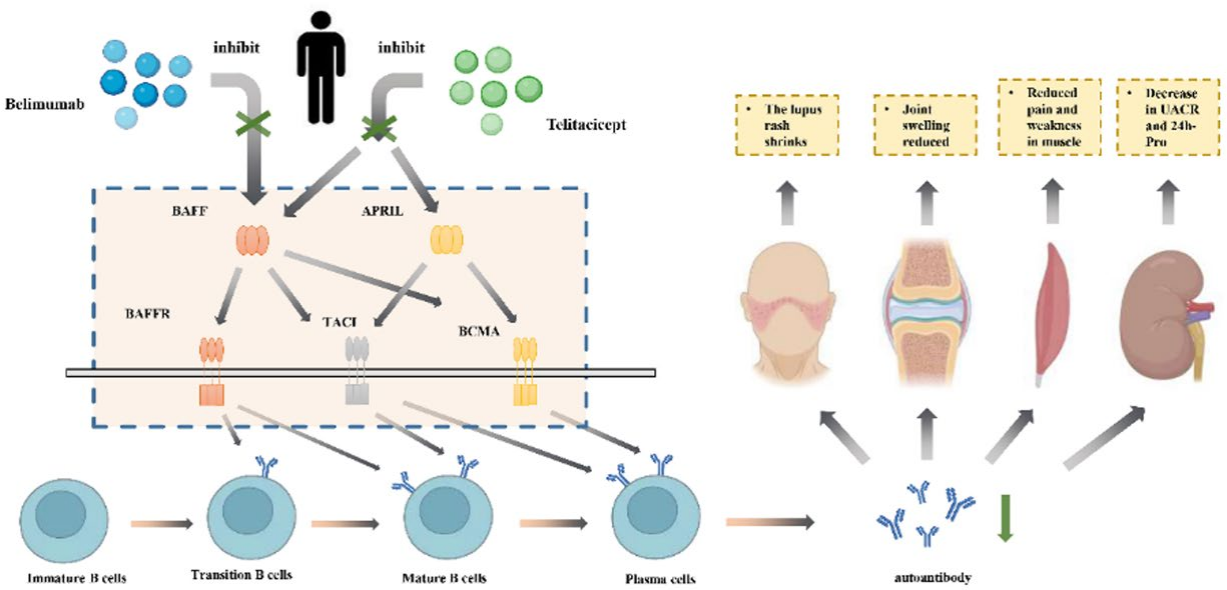
Mechanism of monoclonal antibodies on BAFF and APRIL.

APRIL is a member of the tumor necrosis factor(TNF) superfamily of cytokines and is encoded by the TNFSF13 gene on chromosome 17p13^24^. Previous trials indicated that SLE patients had higher serum APRIL levels than healthy controls, which is positively correlated with SLEDAI scores^25,26^. As shown in Fig. 6, APRIL is produced by several cell types, activated lymphocytes, and mucosal epithelial cells. BAFF is also a key member of the TNF family. It is expressed on the surfaces of monocytes, dendritic cells, and T cells^27^. Of all the TNFSF cytokines, APRIL has the greatest homology to BAFF. APRIL and BAFF share three targets on B cells, including TACI, BCMA, and BAFF receptors. APRIL can bind to TACI together with the BAFF receptor and B cell maturation antigen (BCMA), regulating the function and viability of antigen-stimulated B cells to maintain B cell homeostasis^28^. APRIL binds strongly to BCMA and with lower affinity to TACI. APRIL can also bind heparan sulfate proteoglycans (HSPG) within the extracellular matrix, or on the surface of cells such as plasma cells, increasing its local concentration and activity^29,30^.

Elevated levels of BAFF and APRIL are associated with the pathogenesis of autoimmune diseases. Elevated APRIL levels have been reported in several autoimmune diseases, including SLE, rheumatoid arthritis, sjögren’s syndrome, and multiple sclerosis, and have been associated with more severe disease activity. These two cytokines play overlapping and distinct roles in B-cell proliferation, maturation, survival, and class-switch reorganization. In autoimmune diseases, APRIL and BAFF may promote the survival of self-reactive B cells, which increases autoantibody production^27^. B cells may also influence the extent of tissue damage in disease states by secreting cytokines with anti-inflammatory and pro-inflammatory properties^31^. It has been shown that serum BAFF expression levels in SLE patients correlate with IL-4, IL-6, and IL-10, while APRIL levels correlate with IL-2, IL-12, and TNF-α cytokines^21^. Enhanced B cell depletion may improve the clinical response in SLE patients. Dual blockers may provide more potent and sustained inhibition of pathogenic B cell activity, inhibit the transformation of mature B cells into plasma cells, and promote the apoptosis of long-lived plasma cells by targeting BAFF and APRIL, thus providing the possibility to improve the disease activity in SLE more effectively^22^.

In conclusion, larger, long-term studies are needed to confirm the efficacy and safety of telitacicept. Existing evidence suggests that telitacicept may be superior in the SRI4 response rate. It was also better than the placebo in the SRI7 response rate. It is shown in outcomes of renal function enhancement, prednisone reduction, and anti-dsDNA that telitacicept is not inferior to belimumab. There is no significant difference in AEs. However, the reliability of the result was limited by sample sizes.

## Limitations

We did not have enough direct head-to-head trials, so we used indirect comparison methods. Although we applied strict inclusion criteria and various approaches to assess and reduce bias, our conclusions are likely less reliable than those from direct comparisons. Therefore, further direct head-to-head RCTs are recommended to confirm these findings. The studies included in our review were clinically heterogeneous with respect to patient populations, daily treatment doses, disease duration, and other factors. In the study, we compared two B-cell targeted agents. However, prolonged B-cell depletion increases the risk of infections, and belimumab has been associated with depression and even suicide^32,33^. There are new therapies, other than B-cell targeted therapies, should be paid attention to. We have to minimize the occurrence of adverse events while maximize the clinical benefit.

## Supplementary Information

Below is the link to the electronic supplementary material.

Supplementary Material 1

## Data Availability

The datasets generated during and/or analyzed during the current study are available from the corresponding author on reasonable request.
