# Supplementary material for: Effects of telitacicept and belimumab on systemic lupus erythematosus: a systematic review and meta-analysis
Source: Sci Rep. 2025 Nov 27;15:45506. doi: 10.1038/s41598-025-29929-9 (PMC12748641; doi:10.1038/s41598-025-29929-9)
Supplement: Supplementary file 1 — Supplementary Material 1 [file 41598_2025_29929_MOESM1_ESM.docx]

Supplementary Material

1. Supplementary Data
   1. Search Strategy

PUBMED：

((((((((("telitacicept" [Supplementary Concept]) OR ((Recombinant Fusion Proteins[Title/Abstract]) OR (Telitacicept[Title/Abstract]))) OR ("belimumab" [Supplementary Concept])) OR (((((((((BEL-114333[Title/Abstract]) OR (BEL114333[Title/Abstract])) AND (("Lupus Erythematosus, Systemic"[Mesh]) OR ((((((Lupus Erythematosus, Systemic[Title/Abstract]) OR (Systemic Lupus Erythematosus[Title/Abstract])) OR (Lupus Erythematosus Disseminatus[Title/Abstract])) OR (Libman-Sacks Disease[Title/Abstract])) OR (Disease, Libman-Sacks[Title/Abstract])) OR (Libman Sacks Disease[Title/Abstract])))

Web of Science：

(TS=(telitacicept) OR AB=(belimumab OR BEL-114333 OR BEL114333 OR HGS-1006 OR HGS1006 OR LymphoStat-B OR GSK-1550188 OR GSK1550188 OR Benlysta OR Belimumab) )AND (TS=(Lupus Erythematosus, Systemic) OR AB=(Lupus Erythematosus, Systemic OR Systemic Lupus Erythematosus OR Lupus Erythematosus Disseminatus OR Libman-Sacks Disease OR Disease, Libman-Sacks OR Libman Sacks Disease))

EMBASE:

#1. telitacicept:ab,ti

#2. belimumab:ab,ti OR 'bel 114333':ab,ti OR

bel114333:ab,ti OR 'hgs 1006':ab,ti OR

hgs1006:ab,ti OR 'lymphostat b':ab,ti OR 'gsk

1550188':ab,ti OR gsk1550188:ab,ti OR

benlysta:ab,ti

#3. #1 OR #2

#4. 'systemic lupus erythematosus':ab,ti OR 'lupus

erythematosus disseminatus':ab,ti OR

'libman-sacks disease':ab,ti OR 'disease,

libman-sacks':ab,ti OR 'libman sacks

disease':ab,ti

#5. #3 AND #5

#6. 'randomized controlled trial':it OR 'controlled

clinical trial':it OR randomized:ab,ti OR

placebo:ab,ti OR ('clinical trial':ab,ti AND

topic:ab,ti) OR randomly:ab,ti OR trial:ti

#7. #5 AND #6

Medline:

S4 (PT ( randomized controlled trials or rct or randomised control trials ) OR AB ( randomized or controlled or trials or placebo or randomly ) OR TI trial NOT ( animals not humans )) AND (S1 AND S2 AND S3)

S3 PT ( randomized controlled trials or rct or randomised control trials ) OR AB ( randomized or controlled or trials or placebo or randomly ) OR TI trial NOT ( animals not humans )

S2 AB ( Lupus Erythematosus, Systemic or Systemic Lupus Erythematosus or Lupus Erythematosus Disseminatus or Libman-Sacks Disease or Disease, Libman-Sacks or Libman Sacks Disease ) OR MH Lupus Erythematosus, Systemic

S1 AB Telitacicept or Belimumab or BEL-114333 or BEL114333 or HGS-1006 or HGS1006 or LymphoStat-B or GSK-1550188 or GSK1550188 or Benlysta

https://search.ebscohost.com/login.aspx?direct=true&db=cmedm&bquery=(PT+(randomized+controlled+trials+OR+rct+OR+randomised+control+trials)+OR+AB+(randomized+OR+controlled+OR+trials+OR+placebo+OR+randomly)+OR+TI+trial+NOT+(animals+NOT+humans))+AND+((AB+(Telitacicept+OR+Belimumab+OR+BEL-114333+OR+BEL114333+OR+HGS-1006+OR+HGS1006+OR+LymphoStat-B+OR+GSK-1550188+OR+GSK1550188+OR+Benlysta))+AND+((AB+(Lupus+Erythematosus%2c+Systemic+OR+Systemic+Lupus+Erythematosus+OR+Lupus+Erythematosus+Disseminatus+OR+Libman-Sacks+Disease+OR+Disease%2c+Libman-Sacks+OR+Libman+Sacks+Disease))+OR+(MH+Lupus+Erythematosus%2c+Systemic))+AND+((PT+(randomized+controlled+trials+OR+rct+OR+randomised+control+trials))+OR+(AB+(randomized+OR+controlled+OR+trials+OR+placebo+OR+randomly))+OR+(TI+trial)+NOT+(animals+NOT+humans)))&cli0=FT&clv0=Y&lang=zh-cn&type=1&searchMode=Standard&site=ehost-live

- 1. Supplementary Figures and Tables

**Supplementary Figure 1.** Diagram of judgements about each risk of bias items for eligible trials.


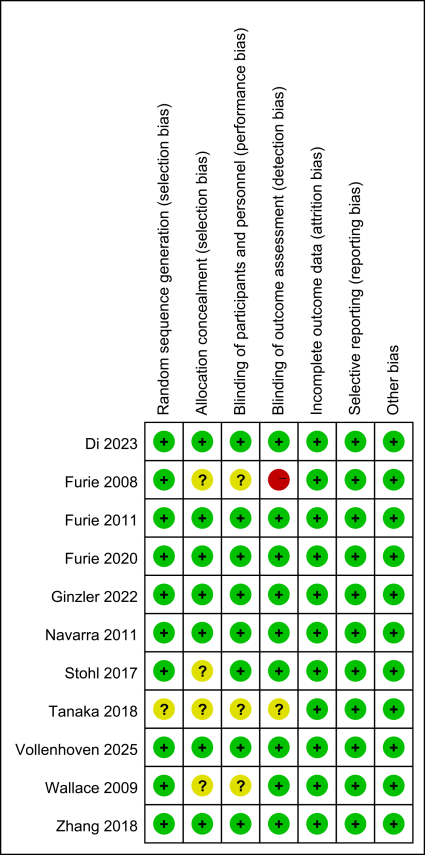


**Supplementary Figure 2.** Diagram conclusion of risk of bias.


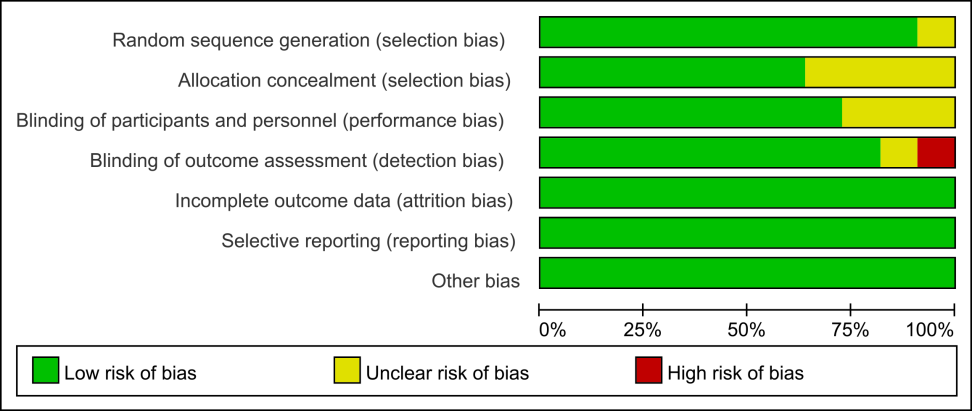


**Supplementary Figure 3.** Diagram of the SRI7 response rate for eligible studies. SRI, the Systemic Lupus Erythematosus Responder Index.


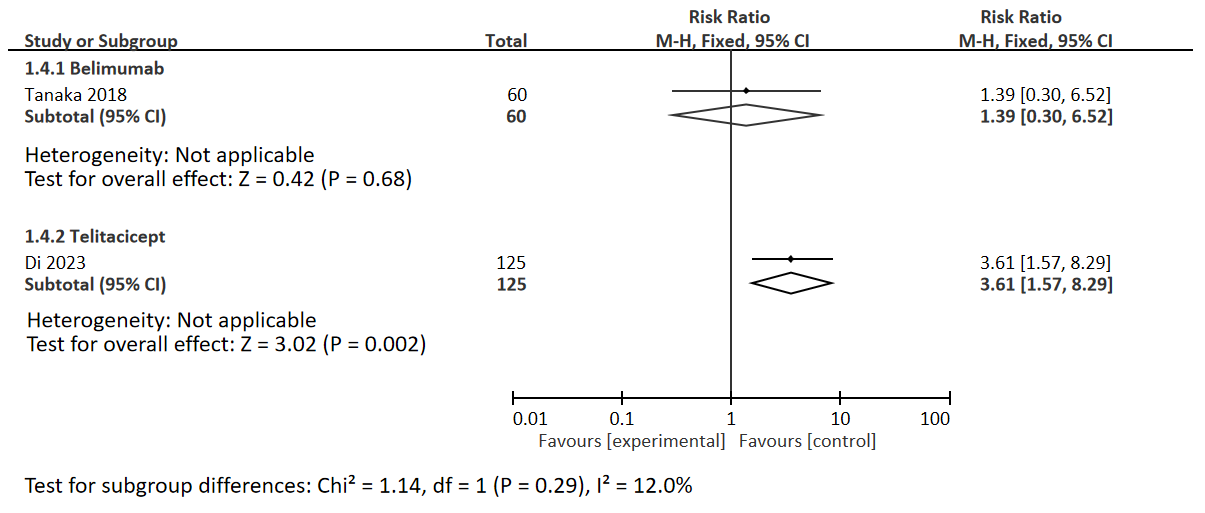


**Supplementary Figure 4.** Diagram of the prednisone dose reduction for eligible studies.

**
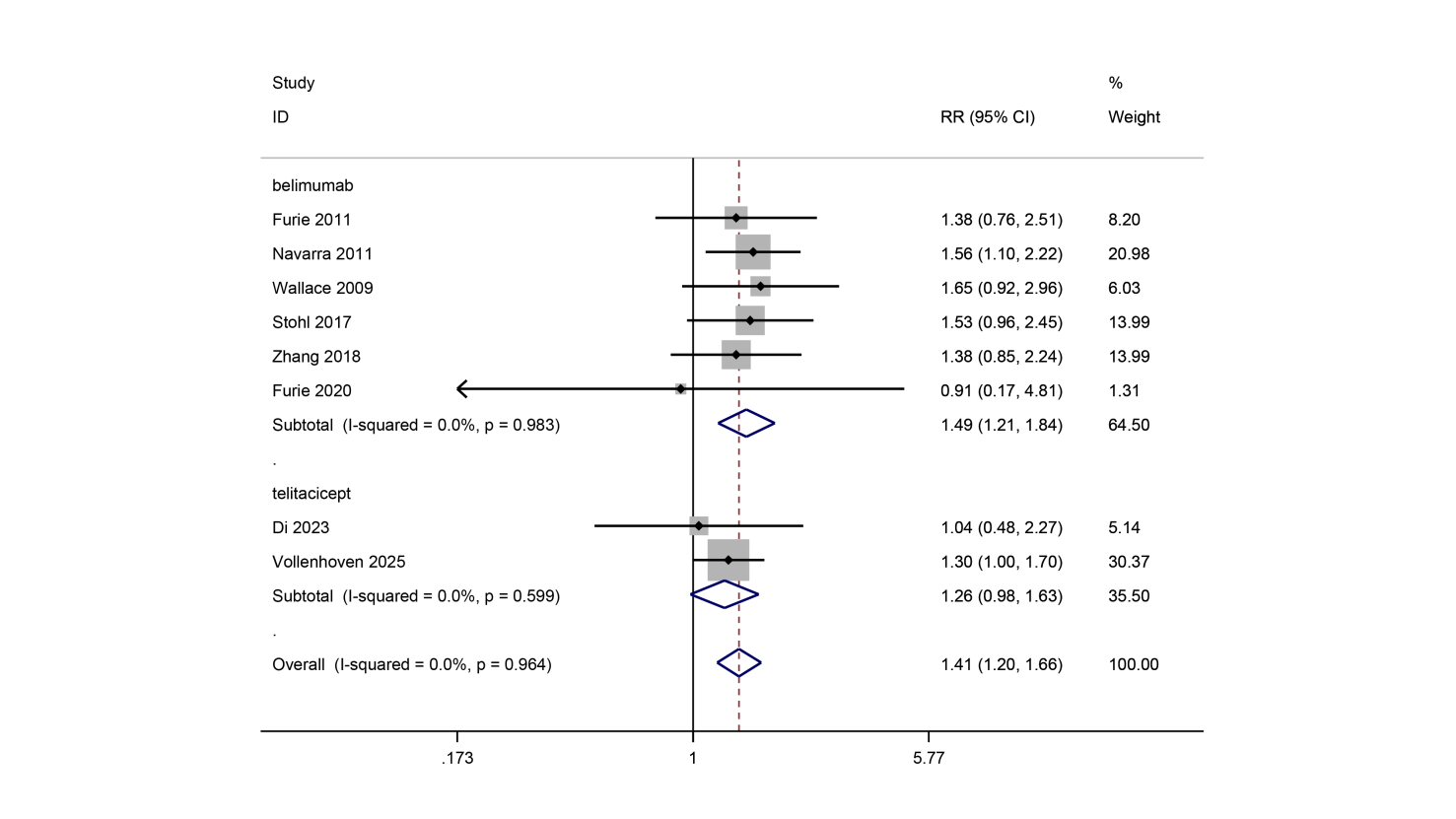
**

**Supplementary Figure 5.** Diagram of anti-dsDNA for eligible studies.


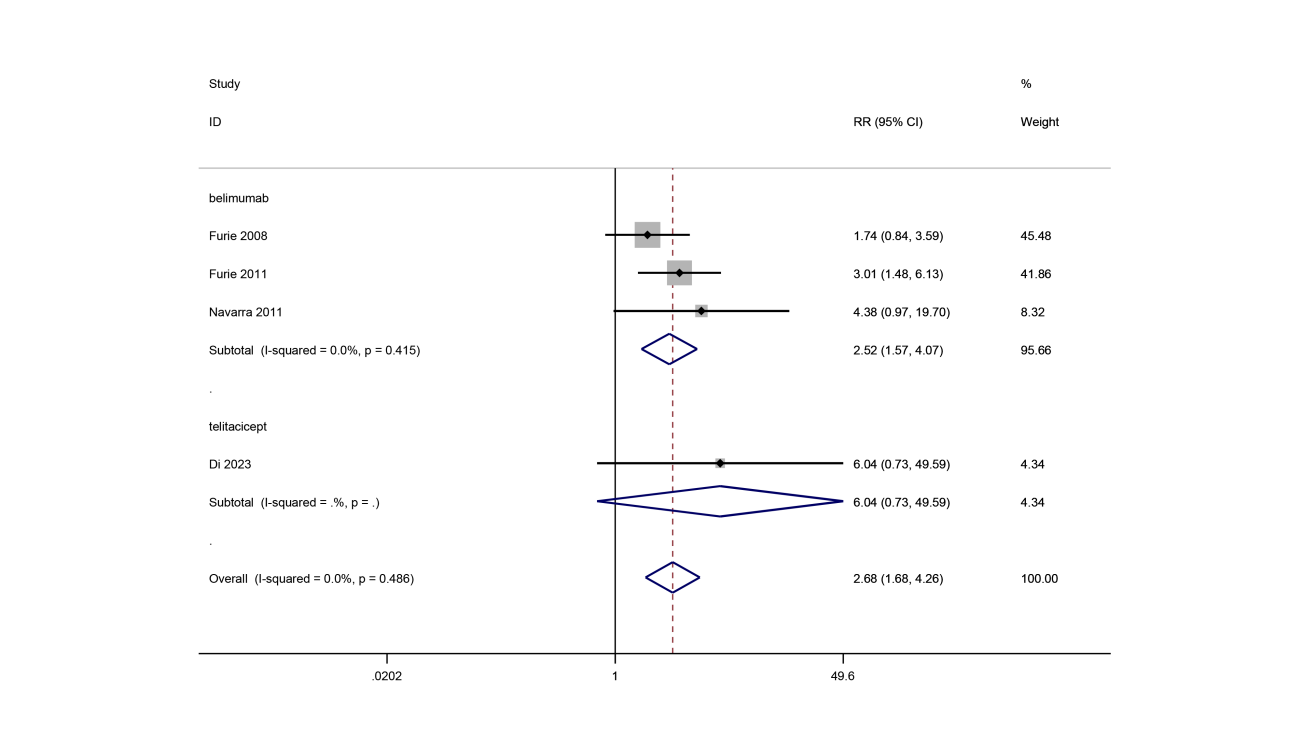


**Supplementary Figure 6.** Diagram of risk of infections and infectious diseases for eligible studies.


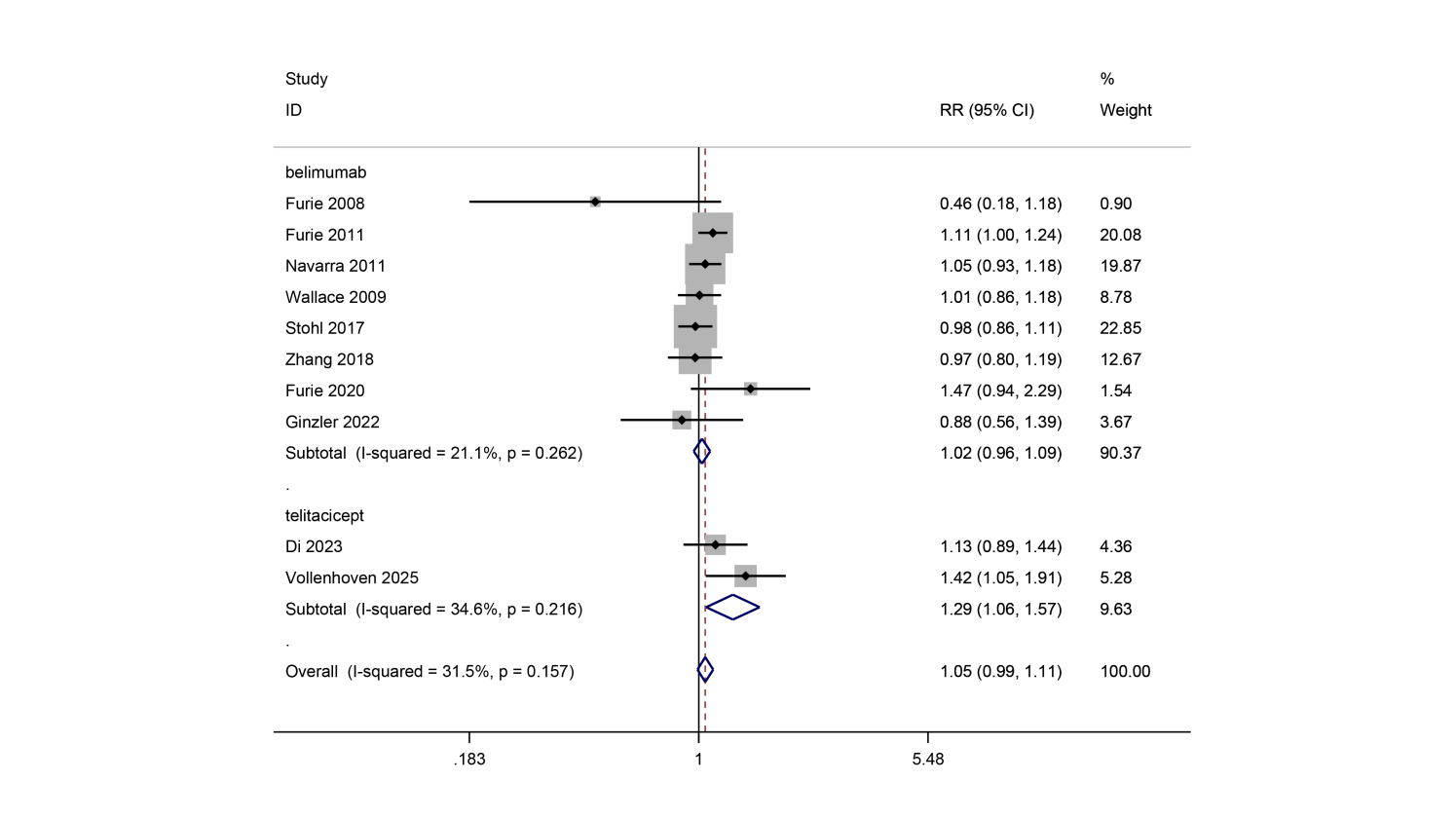


**Supplementary Figure 7.** Diagram of general disorders and administration site conditions for eligible studies.


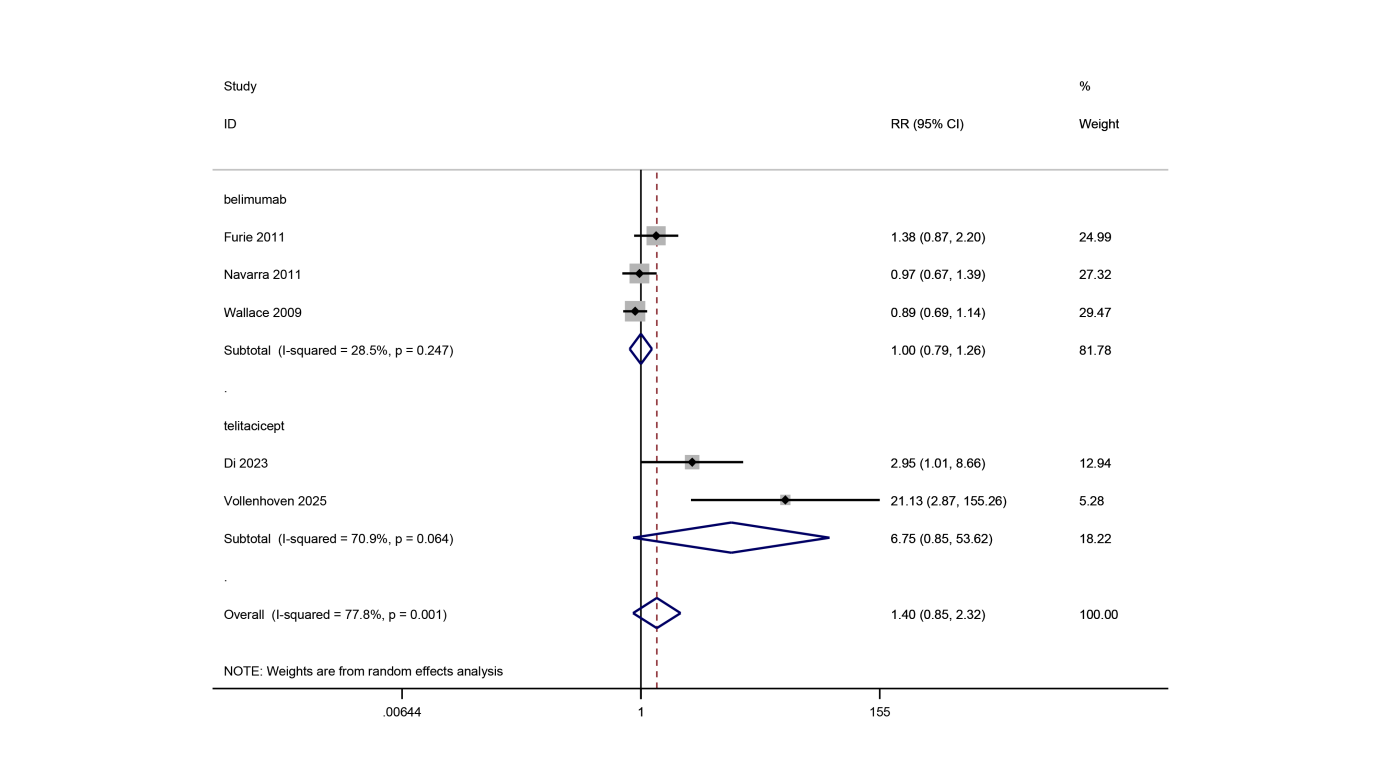


**Supplementary Figure 8.** Subgroup analysis of SRI4 response rate for eligible studies. SRI, the Systemic Lupus Erythematosus Responder Index.

**Supplementary Figure 9.** Publication bias for SRI4 response rate, prednisone dose reduction, AEs and SAEs.

**
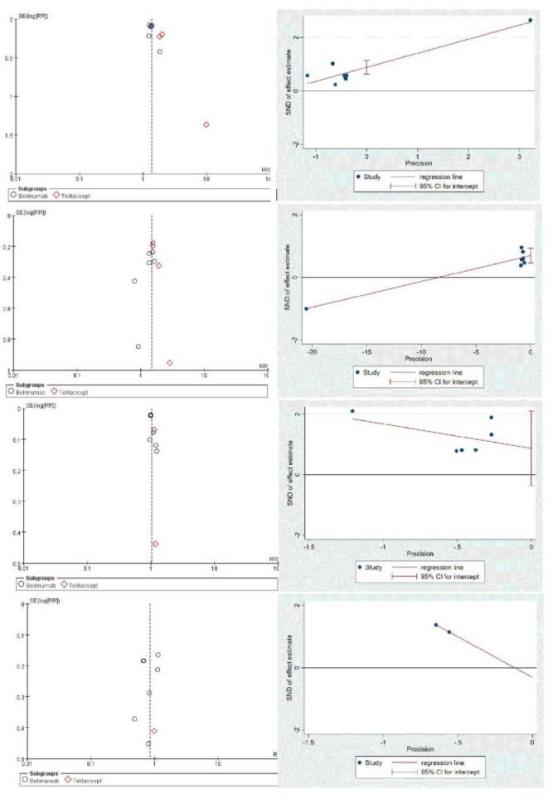
**

**Supplementary Figure 10.** Sensitivity analysis for SRI4 response rate, prednisone dose reduction, AEs and SAEs.

**
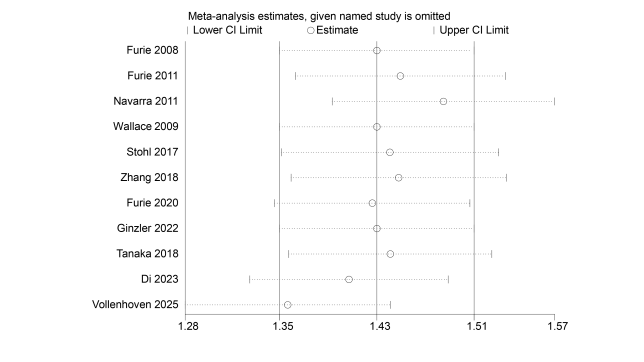

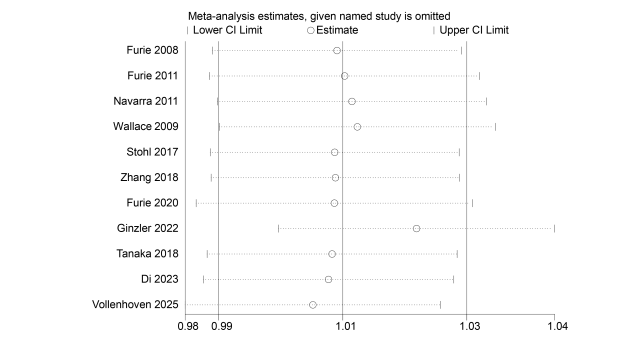

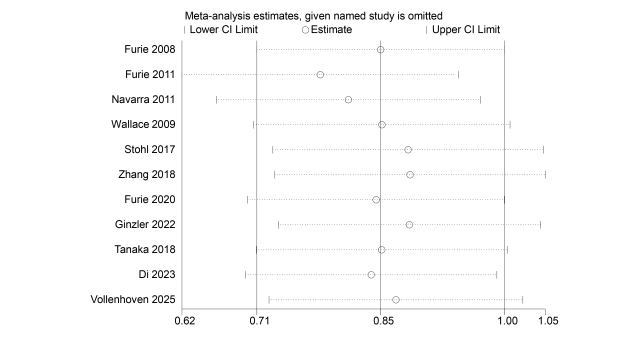
**

**Supplementary Table 1.** Results of ACNOVA analysis.

|  | F score | *p* value |
| --- | --- | --- |
| age | 4.81 | 0.08 |
| gender | 0.37 | 0.56 |
| baseline SLEDAI | 0.61 | 0.48 |
| disease duration | 3.81 | 0.11 |

**Supplementary Table 2.** Results of meta-regression analysis.

| **Covariates (baseline)** | **Dependent** | **Coefficient (95%CI)** | **p value** |
| --- | --- | --- | --- |
| **SLEDAI** | **SRI4 response rate** | **-0.08(-1.48,1.32)** | **0.892** |
|  | **SLEDAI reduction** | **/** | **/** |
| **age** | **SRI4 response rate** | **-0.04(-0.73,0.64)** | **0.886** |
|  | **SLEDAI reduction** | **0.74(-8.09,9.57)** | **0.479** |
| **gender** | **SRI4 response rate** | **-0.29(-4.85,4.26)** | **0.881** |
|  | **SLEDAI reduction** | **0.99(-173.05,175.04)** | **0.954** |
| **injection method** | **SRI4 response rate** | **0.15(-0.07,0.36)** | **0.155** |
|  | **SLEDAI reduction** | **0.19(-15.43,15.81)** | **0.902** |
| **prednisone use** | **SRI4 response rate** | **1.56(-5.63,8.75)** | **0.614** |
|  | **SLEDAI reduction** | **41.53(-414.64,497.69)** | **0.454** |
| **prednisone dose** | **SRI4 response rate** | **0.01(-1.44,1.46)** | **0.988** |
|  | **SLEDAI reduction** | **/** | **/** |
| **disease duration** | **SRI4 response rate** | **-0.15(-0.96,0.67)** | **0.676** |
|  | **SLEDAI reduction** | **2.67(-21.36,26.69)** | **0.393** |
| **follow up** | **SRI4 response rate** | **-0.01(-0.02,0.01)** | **0.674** |
|  | **SLEDAI reduction** | **-0.05(-3.95,3.86)** | **0.902** |
